# The Genetic Basis of Female Mate Preference and Species Isolation in *Drosophila*

**DOI:** 10.1155/2012/328392

**Published:** 2012-08-23

**Authors:** Meghan Laturney, Amanda J. Moehring

**Affiliations:** Department of Biology, The University of Western Ontario, London, ON, Canada N6A 5B7

## Abstract

The processes that underlie mate choice have long fascinated biologists. With the advent of increasingly refined genetic tools, we are now beginning to understand the genetic basis of how males and females discriminate among potential mates. One aspect of mate discrimination of particular interest is that which isolates one species from another. As behavioral isolation is thought to be the first step in speciation, and females are choosy more often than males in this regard, identifying the genetic variants that influence interspecies female mate choice can enhance our understanding of the process of speciation. Here, we review the literature on female mate choice in the most widely used model system for studies of species isolation *Drosophila*. Although females appear to use the same traits for both within- and between-species female mate choice, there seems to be a different genetic basis underlying these choices. Interestingly, most genomic regions that cause females to reject heterospecific males fall within areas of low recombination. Likely, candidate genes are those that act within the auditory or olfactory system, or within areas of the brain that process these systems.

## 1. Introduction

Sex has long been a popular topic of research among evolutionary biologists. Our personal fascination with the subject is related to the variation that is seen in sexual behavior. This includes the different roles that make up mating rituals, such as courtship traits or preference for the traits, and the variation of these behaviors observed both within and between species. Understanding the biological basis of mating behavior is not only interesting, it is also important for our understanding of evolution as it can shed light on how species boundaries are formed and maintained. Different mating behaviors of closely related species can act as an isolating barrier that stops gene flow between two interbreeding populations. This usually results from closely related species having diverse mating signals: one or both of the sexes fail to identify the other as a suitable mate [1–4]. For example, males of some species court conspecific females more often or with more vigor than heterospecific females [5] and females mate more readily with conspecific than heterospecific males [6, 7]. 

The impact of *Drosophila* in this area of research has been pronounced primarily because many obstacles can be bypassed in this system. First, the stereotypical mating behavior observed in this genus is relatively easy to score [8–10], there are genetic tools available to allow manipulation of the development and physiology of mating behavior [11], and there is relative ease in housing large numbers of individuals in a uniform environment. Second, many *Drosophila *sister species are only partially isolated in a lab setting, producing viable and fertile hybrids [2].

Females of most *Drosophila* species are usually the sex that determines whether copulation occurs [9]. Males preferentially court conspecific females with larger body sizes, which is a good indicator of female fecundity [12], and in some species (e.g., *D. virilis*) males are able to discriminate against heterospecific females [5]. However, it is more often found that males readily court heterospecific females [13], while females discriminate against heterospecifics males [6]. Females easily prevent unwanted copulations by flying away from the courting male orextruding her ovipositor [8]. Furthermore, mating behavior has been found to be cyclic with alternating bouts of high mating activity and low mating activity; with the use of arrhythmic mutants, it has been shown that females determine when mating occurs [14]. Therefore, in order to understand what isolates species from each other, attention should be focused on female mating behavior.

## 2. The Evolution of Genes for Female Mating ****Behavior

The majority of research in behavioral isolation has been influenced by the Modern Synthesis [15], which is a general account of speciation and evolution. The tenants of the Modern Synthesis state that a population contains genetic variation which is apparent at both the gene level (with multiple alleles produced by random mutation) and at the chromosomal level (with different combinations of alleles within individuals produced by recombination). A population's gene frequencies and allele combinations can change over time through multiple processes, including natural selection and genetic drift. While the contribution of natural selection has been wellsupported, the impact of genetic drift has been debated within the literature. For example, a computer model exploring the development of behavioral isolation via sexual selection [16] and research that employed extreme bottlenecking [17] both showed that genetic drift can rapidly lead to some level of sexual isolation. On the other hand, speciation by genetic drift has been shown to be unlikely to occur [18, 19] because genes for mating behavior are most likely either pleiotropic and directly under natural selection, or are closely linked to genes that are under selection and therefore would not simply be fixed by a random process [20].

If a population is divided, the newlyformed subpopulations can potentially become genetically differentiated from each other. Over time, the genomes of each subpopulation can diverge from each other either due to the different distribution of alleles that made up the founder population, the different selective pressures on these alleles, and new genetic mutations that arise. As with other traits, the genetic variants that contribute to male and female mating behaviors may cause a difference in phenotype between individuals of the two subpopulations (for review, see [21]). Differences in mating signals can influence female mate choice, which can subsequently act to reduce gene flow between the groups if they come into contact. 

Secondary contact between diverging populations can, however, produce hybrids between species in nature. If these hybrids have a relatively high fitness, it is possible that enough gene flow can occur between these two species to cause them to merge back into one. In contrast, if hybrids have a low fitness, a selective pressure to assortatively mate within both populations can act directly on the genes for mating behavior, favoring alleles that differentiate courtship behaviors and enhance preferences for traits that distinguish potential mates of the two groups. This phenomenon, known as reinforcement, has been observed in nature where two closely related species, for example, *Drosophila pseudoobscura *and *D. persimilis*, have partially overlapping regions. In response to the selective pressure to avoid heterospecific matings, populations from the sympatric region have a greater level of behavioral isolation compared to those from allopatric populations [22, 23]. However, reinforcement's role in speciation was historically disputed as alternative theories could explain the increased level of behavioral isolation [24], controlled experiments on the topic were generally lacking [25], and some experiments failed to support the theory of reinforcement. For example, the presence of reinforcement within *D. mojavensis* and *D. arizonensis* was tested with the use of two groups: one with the traditional rearing substrate of banana agar food, and the other with fermenting cactus—the natural food of these two species. Although behavioral isolation was still found between the species, and the general pattern of reinforcement was still present, the sympatric population was not significantly more behaviorally isolated than the allopatric population [26]. Additionally, reinforcement is not required for differences in mating behavior to arise. For example, a population of *Drosophila *was subdivided into three groups within the lab: one group remained on the ancestral food source, while two other subpopulations were reared on novel food sources for multiple generations. Afterwards, female mating preferences were tested and were found to be changed in parallel with population divergence [27]. 

Recently, however, strong empirical data in support of reinforcement has surfaced. Lab investigations have shown that reinforcement can strengthen behavioral isolation between two closely related species [28], and once the selective pressure for species discrimination is removed, the likelihood of interspecific mating has been found to increase [29]. A meta-analysis also found evidence to support the previouslyuntested predictions of reinforcement, such as concordant isolation asymmetries (because reinforcement potentially evolves due to unfit hybrids, pre- and postzygotic isolation should evolve in the same direction) and rare-female effect (females from the smaller population would encounter more heterospecific males and therefore have a stronger selective pressure to choose conspecific males) [30]. Thus, separated populations can evolve divergent behaviors, and these behaviors can potentially be enhanced when the populations are once again in contact.

## 3. Intraspecific Sexual Selection versus ****Interspecific Female Mate Choice

The relationship between within- and between-species mating preferences is not fully understood, but they are often considered extreme ends of the same continuum. With time, two populations of a species are thought to slowly slide from assortative mating to heterospecific discrimination, by sexual selection either directly acting on genes that influence mating behavior or indirectly acting on genes that enhance survival. Blows and Allan [1] argued that if species isolation was produced by sexual selection, then the traits involved in species isolation should be the same traits used by both sexes during within-species mate choice. To test this hypothesis, they investigated the behavioral isolation between *D. serrata* and *D. birchii*. These two species have overlapping geographic regions along the east coast of Australia. Although morphologically very similar, there is strong behavioral isolation between the two species [31]. They showed that the two species have different cuticular hydrocarbon (CHC) profiles, which are used as sexual pheromones. By performing perfuming experiments, which transferred CHCs from one species onto another, these researchers determined that the same mechanism (olfaction sensation of CHCs) is used for within-species mate choice (sexual selection) and between-species female mate preference (behavioral isolation). 

Although this shows that variation in the same trait can be used for both within- and between-species female mate choice, it does not necessarily mean that they have the same genetic basis. The assumption is that there is a set of genes that control female mating behavior in the ancestral population, and once the population is divided, those genes accumulate mutations in the new populations which cause changes in the behavior. The genes that control intraspecific behavioral variation, however, may not be the same genes that are important in interspecific behavioral variation. Although, for example, genes for olfactory system development used to detect different CHC profiles could be important for normal female mating behavior in both species, the genetic basis for the interpretation of variation in the CHC profile may vary between species. 

Investigation into this question led to a series of studies that showed the relationship between interspecific hybridization and intraspecific receptivity. Carracedo et al. [32] proposed that if intraspecific and interspecific mating behaviors have the same genetic basis, females that are slower to accept conspecific males may also be more reluctant to accept heterospecific males. In other words, high level of general within-species receptivity would be selected against due to its pleiotropic effect on high interspecies hybridization. In the lab, when a high level of interspecies hybridization (reduced choosiness) in females was selected for, a decrease in time to start copulation with conspecific males was also found [33]. This was interpreted as a linked increase in intra- and interspecific receptivity, giving support to the notion that both types of mating behavior have the same genetic underpinning. However, when interspecific mating was directly tested by placing females in a choice assay with conspecific and heterospecific flies of the opposite sex, almost no heterospecific matings were observed, showing that selection for heterospecific mating is unlikely to influence within species mate choice in nature, where multiple mates are available [34]. The ultimate test of whether the genetic bases of intra- and interspecific mating behavior are under the same genetic control would be to determine and compare the genetic basis of both systems. Unfortunately, no gene has been identified to be involved with interspecific female mate choice. However, a few studies that have identified the regions that most likely contain genes that isolate species do not seem to overlap those regions that contain genes known to influence within-species mating behavior [6, 7, 35].

More unexpected results came from the female mating behavior of island populations. When migrants populate a new island, it is likely that the least choosy females will propagate the most offspring since the most choosy females may not find a high-quality male and therefore will not reproduce [36, 37]. Assuming that low intraspecific choosiness results in high hybridization rates, we would then expect isolated island species to have high levels of hybridization. Although we do find this relationship in the North American and Bogota strains of *D. pseudoobscura* [38], we see the opposite trend in many other species pairs [19]. For example, *D. mauritiana* and *D. sechellia* females, both from island populations, are more choosy against males from the closely related mainland species, *D. simulans*, than mainland females are against island males.

## 4. Genes for Interspecific Female Mating ****Behavior

Mating behaviors in *Drosophila* usually have a genetic basis (e.g., of an exception, see [39]). The genetic information that one inherits can predispose a female to behave a specific way: which partner she chooses to accept. These genetic factors can influence both behavioral variation within a species and behavioral differences between species. The latter of these two is critical for our understanding of the genetic basis of species isolation, as it is thought that these behavioral differences are the first barrier to arise in species isolation [40]. By identifying the genetic variants that cause interspecific differences in mating behavior, we can determine which mutations and alterations in the genetic material cause the differences in behavior between two isolated species, and thus may underlie the speciation process itself. 

Despite its importance for species isolation, the genetic basis of behavioral isolation is not well understood. This is primarily due to the most commonly used method in genetics for locating genes that contribute to variation in a quantitative trait, namely, recombination mapping. This method necessitates crossing two divergent lines and producing fertile offspring. However, by definition, separate species usually do not produce either fertile or viable offspring. Second, identifying the genetic basis of a behavior requires the location of multiple genes with different effect sizes [41], necessitating a repeatable measure of the behavior, large sample sizes, and the availability of powerful genetic tools such as readily available single gene mutant lines [42].

Despite these obstacles, the genetic basis of mating behavior has been studied in different species of animals and plants. The genetic basis of floral scent production in *Petunia axillaris* (*Petunia*) has been found to play an important role in pollinator attraction and thus contributes to isolation between related species of plants [43]. Research on butterfly mating behavior has found a consistent relationship between wing color and mate preference [44] and both traits may be caused by the same gene (*wingless*) or multiple genes linked to *wingless* [45]. Male cichlids in Lake Victoria have divergent species-specific coloration which has been shown to be driven by female choice [46] and this interspecific female mating preference for conspecific coloration has been found to be heritable in cichlids, with only a few loci responsible [47]. Although butterfly and cichlid coloration and preference have provided insight into the genetic basis of behavioral isolation, these systems are limited in that they do not have the powerful genetic tools that are available in *Drosophila*, a well-developed genetic model system. 

Using mutagenesis studies, multiple genes have been identified in *D. melanogaster* that influence within-species mating behaviors for both males and females. Male behavior has traditionally taken the spotlight in genetic studies on mating behavior. Through mutagenesis studies, approximately 55 genes have been identified to influence within species male mating behavior, while only a handful of genes have been identified that act within a female to increase or reduce her receptivity (see Supplementary Table 1 available online at doi:10.1155/2012/328392).

These studies are of great importance as they provided crucial information into both the sensory system used in *Drosophila* mating and the types of genes that can influence the construction of mating behavior. However, these studies eliminate the gene's function in order to test whether it affects a behavior. While this demonstrates that the gene is important for creation of the behavior, it does not necessarily tell us anything about the naturally occurring genetic variation that contributes to the differences seen within or between species. For example, genes identified during mutagenesis for normal male mating behavior were not found to contribute to variation seen in courtship [48], did not contribute to variation between low and high mating male lines [41], and did not vary in expression in a natural population of *D. melanogaster* [49]. The genes important for normal female mating behavior were also not found to vary in expression between courted and naïve same-age virgin females [50]. The genes identified through mutagenesis consistently do not appear to influence the variation in mating behavior within a species, and, therefore, may also not contribute to the variation observed between species [51].

Although no individual genes for behavioral isolation have been identified, recombination mapping studies have located regions of the genome that influence behavioral isolation, which do not include genes identified through mutagenesis (see below). However, since the preliminary observations of interspecific female mating behavior do not resemble the expectations set out by prevailing theory, it is difficult to determine strong candidate genes for interspecific female receptivity within these regions [52, 53]. In order to identify which genes are candidates for influencing interspecific female mating behavior, we could first evaluate which signals females are basing their choice upon.

## 5. The Modes of *Drosophila* Male Signaling ****during Courtship

The variability we see in female preference, both within and between species, is most likely dictated by the integration of the auditory and olfactory systems [54]. To complicate investigation of these two systems, the amount that females of each species rely on one system over the other is most likely species specific [3, 4, 55, 56]. A gene for interspecific female preference is most likely going to be associated with the signaling pathway of the auditory system used to recognize differences in male courtship song characteristics [3, 57], the olfactory system used to recognize CHC pheromone profiles [1], or both systems via the organization of the part of the brain that receives and interprets signals from both pathways [54, 58]. This is because both modes of signaling are used during *Drosophila* courtship [8–10] and vary between species [1, 3, 56, 59]. A candidate region for such integration in the brain is the mushroom body, which receives signals from many sensory systems in *Drosophila* [60], including the olfaction system [61], and has been linked to sexual behavior [62, 63], specifically female receptivity [64]. 

There are two main elements to the courtship song—the sine song and the interpulse interval—and males of different species usually differ from each other on both accounts [53]. A female's ability to identify conspecific song over heterospecifics can lead to behavioral isolation [3]. For females in the melanogaster group of *Drosophila*, the most important element of courtship song is the interpulse interval (IPI) which differs among the males, and preference for variants of IPI seems to differ among females [65]. The most famous gene to influence courtship song is the *period *(*per*) gene. Mutations in this gene influence IPI [66], and transgenic *D. melanogaster* flies with *D. simulans per* produced *D. simulans*-typical rhythm [57]. Instead of a species difference reflecting a complex genetic basis, the species differences in song rhythm reflect just a small number of amino acid changes [57]. Females from this same transgenic line showed associated preference for the transgenic male's IPI [67], and a later study also showed evidence of assortative mating with a different *per*-transgenic line [68]. Although the genetic basis of this preference is not straightforward, it is clear that females may be using the variations in song between species in determining mate choice. Females can detect male song and male movement with use of the receptors in the antenna; neurons from the antenna project to the dorsal brain, which requires feminization in order for females to be receptive (for review, see [58]). 

In addition to song, females also use pheromonal cues to distinguish mates. Each species of *Drosophila* has cuticular hydrocarbons (CHCs) on the outer surface of their body that act as a protective barrier to desiccation and most likely evolved as an adaptation to dry climates [69]. These compounds also are important in mating behavior [70] and are used during mate selection as pheromones that both allow males to distinguish females [71] and affect female receptivity [72]. The majority of CHCs are nonvolatile compounds that are detected by both males and females, most likely through touch (gustation) at close proximity, rather than smell at long distances [70]. Detection of the CHC profile occurs through a large family of odorant receptors that send information about the environment via odorant sensory neurons to the antennal lobe, which is analogous to the olfactory bulb in mammals (for a review, see [58]). 

Billeter et al. [71] used a Gal4-UAS system to block the development of oenocytes, which are cells specialized to produce the cuticular hydrocarbons. Flies without working oenocytes (oe^−^) were completely devoid of all CHCs but behave normally. However, female response towards oe^−^ males was significantly altered: wildtype females were significantly less receptive to oe^−^ males and oe^−^ males took significantly longer to achieve copulation. Therefore, CHCs not only enhance within species female receptivity [71], but they can also potentially be used to deter females from heterospecific matings [1]. Furthermore, it has been shown that males' CHC profiles respond more easily to lab-induced natural and sexual selection than the females' CHC profile [73], indicating that the male profile could be a more likely avenue by which selection acts in nature. 

Although there are more than 20 different CHC molecules on the cuticle of the fly, only the predominant hydrocarbons have received much examination and have been primarily studied within the melanogaster subgroup of *Drosophila* [74]. *D. simulans *and *D. mauritiana* have a monomorphic CHC profile, with the main hydrocarbon of both males and females being the same 23-carbon chain compound, *cis* 7-trisosene (7-T). However, *D. melanogaster *and *D. sechellia* are dimorphic: the males have large amounts of 7-T, but females lack this hydrocarbon and instead have large amounts of a 27-carbon molecule, *cis, cis*-7,11-heptacosadiene (7,11-HD) [75]. Most *Drosophila *species have males that predominately produce 7-T as their main CHC and also share multiple minor compounds as well. However, the ratio between the different CHCs is slightly altered between species, creating unique pheromone “blends” [70].

Through mutagenesis studies, genes have been identified to affect CHC production, such as *dsat1* and *dsat2* [76], *Enhancer of zest* [77], *Ddc* [78], *nerd* [79], *seven pentacosene*, and *smoq *[80], as well as some sex determination genes, such as *doublesex* [81]. However, only the genetic basis of the main CHC components (7-T and 7,11-HD) have been examined. Additionally, it is unclear if variation in these genes produces the variation that is seen in CHC production between populations of the same species, or variation in production between species [56, 82, 83].

 From the research dedicated to identifying the genetic basis of CHC variation between species and courtship song variation between males of different species, we can comfortably deduce that different species have different CHC profiles, different courtship songs, and females preferentially mate with conspecific males based at least partially on both signals.

## 6. Genetic Basis of Female Behavioral Isolation for Different Species Pairs

To date, no individual genes have been identified as influencing intra- or interspecific female preference in *Drosophila*, although the trait has a clear heritable basis [8]. Due to the requirement of fertile hybrids for traditional recombination mapping, the majority of studies seeking to address this question have been done in *Drosophila *species other than *D. melanogaster* (Table 1), since *D. melanogaster* does not produce fertile offspring with any of its sibling species[2, 3, 6, 7, 59, 84]. The majority of studies that have examined the behavioral isolation between *D. melanogaster* and *D. simulans* have done so in a limited way, showing that specific chromosome arms influence behavioral isolation, and until recently these attempts have not come close to isolating individual genetic variants that affect behavioral isolation [85–87]. However, the genomes of 12 different species of *Drosophila* have now been sequenced [88], and recently the powerful genetic tools available in *D. melanogaster*, such as the Gal4-UAS system (used to manipulate gene expression) and transposon vectors (for use in mutagenesis studies), have now been modified for other species of *Drosophila* [89]. Despite the previous limitations, various genomic regions have been identified that contribute to behavioral isolation in multiple species of *Drosophila*, and the expansion of the available tools makes further refinement of these studies now possible.

### 6.1. * D. pseudoobscura * and * D. persimilis*

*Drosophila pseudoobscura* are found across much of Western North America and are located both in sympatry and in allopatry with *D. persimilis* [123]. The initial genetic basis of isolation between these species, termed basal isolation, was found to be caused by only two regions in the genome: one on the left arm of the X chromosome (which is homologous to the X in *D. melanogaster*) and one on the second chromosome (homologous to the right arm of chromosome 3, called 3R, in *D. melanogaster*), within an interspecific inversion that differentiates *D. pseudoobscura* and *D. persimilis* [84].

Female *D. pseudoobscura* from sympatric regions hybridize less with male *D. persimilis* than females from allopatric regions without *D. persimilis*, which has made this a model system for studying reinforcement [22]. Ortiz-Barrientos et al. [109] investigated the genetic basis of the increased discrimination of sympatric *D. pseudoobscura *females. By introgressing (crossing) pieces of the sympatric *D. pseudoobscura* genome into an allopatric *D. pseudoobscura* background, they mapped the increase in behavioral isolation to two alleles of strong effect, one on the right arm of the X chromosome (called *Coy-1*; homologous to 3L in *D. melanogaster*) and one on the fourth chromosome (called *Coy-2*; homologous to 2L in *D. melanogaster*). However, Barnwell and Noor [124] used six pairs of different inbred strains in a quantitative trait locus (QTL) mapping study to try to replicate the previous identification of *Coy-1* and *Coy-2*. They could not, and therefore determined that *Coy-1* and *Coy-2*, although they may be important, are not the primary loci causing increased behavioral isolation in sympatric versus allopatric populations. These alleles may be present at low frequencies in natural populations and therefore would not be present in most inbred laboratory lines.

Although they may not underlie species-wide discrimination, an examination of the two loci could still provide important insight into the genetic basis of reinforcement. To this end, each of the *D. pseudoobscura* sympatric and allopatric *Coy2* alleles was introgressed into a* D. persimilis* background (creating per*Coy2*sym and per*Coy2*allo lines) [110]. If the reinforced behavioral isolation was caused by an increased receptivity for *D. pseudoobscura* (conspecifics) by the *D. pseudoobscura* sympatric population, the expected results would be that per*Coy2*sym females are more likely to mate with *D. pseudoobscura* than per*Coy2*allo, but instead they found the opposite: per*Coy2*sym females were less likely to mate with *D. pseudoobscura* than per*Coy*2allo. This suggests that an allele for reduced interspecific mating within a species (*Coy2*sym) can cause the same reduction in interspecific mating when placed within another species [110]. The explanation provided by Ortíz-Barrientos and Noor is that *Coy-2* may be a “One-Allele” mating locus. This theory suggests that one allele (*Coy-2*) can exist in both the sympatric population of *D. pseudoobscura* and in *D. persimilis* population, and aids in the reinforced behavioral isolation between these populations, but not in the basal behavioral isolation. In other words, the same allele causes females of both species to have an increased discrimination against heterospecifics. This is possible if, for example, the gene encodes for increased odor sensitivity or reduced dispersal [125]. This theory would explain why per*Coy2*sym females were less likely to mate with *D. pseudoobscura* than per*Coy2*allo. 

### 6.2. * D. ananassae * and * D. pallidosa*

*Drosophila ananassae* and *D. pallidosa* are present in overlapping pan-tropical geographic regions. Males of both species court females of both species, but there is strong female interspecific female preference that reduces the gene flow between the two. The genetic basis of this behavior was first explored with female F_1_ hybrids, which were found to prefer *D. ananassae* males over *D. pallidosa* males [3]. This suggests that *D. ananassae* genes for interspecific female choice must be dominant over those from *D. pallidosa*. The same study created introgression lines to locate the genomic regions responsible for this behavior. A region on the left arm of the second chromosome (homologous to 3R in *D. melanogaster*) near the *Delta* locus was identified to play a role in female species mate choice: females that were almost entirely *D. pallidosa* except for a small region near the *Delta* locus mated significantly more with *D. ananassae* males and significantly less with *D. pallidosa* males [3]. In other words, this locus both increased intraspecific mating in *D. ananassae* and decreased interspecific mating between *D. ananassae* females with heterospecific males. This region was later confirmed by a study that found 2L (3R in *D. melanogaster*) to be important for the willingness of *D. pallidosa* females to mate with *D. ananassae* males, and XL, 2L, and 3R (X, 3R, and 2L in *D. melanogaster*, resp.) for *D. ananassae* female's willingness to mate with *D. pallidosa* males. All of the identified regions had species specific inversions [121], suggesting that regions of the genome with reduced recombination between the species may be more likely to harbor behavioral isolation loci.

### 6.3. * D. santomea * and * D. yakuba*

*Drosophila santomea* and *D. yakuba* diverged approximately 400,000 years ago [126]. *D. yakuba* is wide-spread across Africa, including some of the islands off of the coast. On one of these islands, *D. santomea* are found [127]. Although this species pair has a small overlapping geographic region, no reinforcement has been observed [128]. Male courtship behavior may contribute to the behavioral isolation between these two species as *D. santomea* males do not court heterospecific females with any vigor. To investigate the genetic basis behind the female interspecific mating, a QTL map was created for female rejection of heterospecific males [7]. Three QTLs were identified for *D. santomea* female discrimination against *D. yakuba* males: two on the X chromosome (homologous to X in *D. melanogaster*) and one on the third chromosome (3R in *D. melanogaster*).

### 6.4. * D. simulans * and * D. sechellia*

*Drosophila simulans *is a cosmopolitan species, while its closely related sibling species *D. sechellia* is only found on the Seychelles Islands in the Indian Ocean. There is an asymmetrical behavioral isolation between *D. simulans* and *D. sechellia*: *D. simulans* females are less choosy against *D. sechellia* males than *D. sechellia* females are against *D. simulans* males [2]. Hybrids have an intermediate level of *D. simulans *rejection when paired with *D. simulans* males, suggesting an additive genetic basis. Further backcrossing of these F_1_ hybrids to *D. simulans* males, and pairing the female offspring with *D. simulans* males, revealed no isolation, and therefore locating the genes for behavioral isolation in *D. sechellia* females is not possible with this technique. When the F_1_ hybrids are backcrossed to *D. sechellia* males, and the resulting females were assayed with *D. simulans* males, the second and third chromosomes (2 and 3 in *D. melanogaster*) were found to have a moderate and strong effect, respectively [2]. 

### 6.5. * D. simulans * and *D. mauritiana*

*D*. *simulans* is a cosmopolitan species and *D*. *mauritiana* is only found on the island of Mauritius in the Indian Ocean. It is thought that *D*. *mauritiana* resulted from colonization by a recent common ancestor with *D*. *simulans* about 250,000 years ago [129]. Females of these species are almost identical, and the males are only distinguishable by the shape of their genital arch [130]. Asymmetrical species isolation is present, with *D*. *simulans* being the less choosy of the two courted females. Although *D*. *simulans* females are not choosy and readily mate with *D*. *mauritiana* males, matings between these two species are abnormally short and result in no or limited sperm transfer, decreasing the number of hybrid offspring [2].

The absence of heterospecific mating by *D. mauritiana *females is due to the rejection of males by these females, since females of both species are courted vigorously by males of both species [13]. Hybrids produced by *D. mauritiana* males and *D. simulans* females mate readily with *D. simulans* males, and thus the genes for interspecific mate discrimination in *D. mauritiana* females must be recessive [2, 13]. By backcrossing the hybrids to *D. mauritiana *males, Coyne was able to asses each *D. mauritiana* chromosome's effect on decreasing mating with *D. simulans* males [13], He found each of the main autosomes has very large effects with the effect of X being very small [13]. Further dissections of the second chromosome determined that each arm of the second chromosome contains at least one gene for reducing *D. mauritiana *f*e*male mating with *D. simulans* males (2R and 2L in *D. melanogaster*); this method of uncovering recessive *D. mauritiana* genes also possibly removed *D. simulans* genes for conspecific mate preference—these genes may or may not be one in the same. When the same pairings were examined with a more refined map, seven QTL were identified that contribute to *D. mauritiana* discrimination against *D. simulans* males: two on the X chromosome, two on the second chromosome, and three on the third chromosome (X, 2, and 3 in *D. melanogaster*, resp.) [6]. 

### 6.6. *D. simulans * and *D. melanogaster*

*Drosophila melanogaster* and *D. simulans* are both cosmopolitan species found worldwide and have broad overlapping geographic distribution. Although both females show some behavioral isolation, *D. simulans* females are far more choosy [131, 132]: interspecific crosses with *D. melanogaster* females are produced with relative ease in the lab, but the reciprocal interspecific cross with *D. simulans* females very rarely occurs [133]. F_1_ hybrids made from* D. melanogaster *females are all sterile females, and from the reciprocal cross are all sterile males. Due to the complete sterility of hybrids, the conventional method of QTL mapping is not possible as this would require an F_2_ generation, typically through backcrossing to one of the parental species. Therefore, other methods used to determine the genetic basis of behavioral isolation between these two species have been employed.

Using chromosomal substitution, a genomic region was identified on the third chromosome for *D. melanogaster *female receptivity, and genomic regions on all three major chromosomes were identified for rejection of *D. simulans* males by *D. melanogaster* females [85]. Although there is some evidence that male *D. simulans* may have reduced courtship of interspecific females, and thus contribute to the behavioral isolation [132], there is no such evidence for discrimination by *D. melanogaster* males [134]. Therefore, the strong behavioral isolation demonstrated by *D. simulans* females is largely due to rejection of heterospecific (*D. melanogaster*) males. 

To investigate whether there is genetic variation for *D. simulans *female preference, different lab strains of *D. simulans* females [86, 135] and *D. melanogaster* males [86] were compared for their rate of interspecific mating. Crossability, the ability for the parental strains or species to successfully produce offspring, varied among strains for both *D. melanogaster* males and *D. simulans* females [86, 87], but were still highly correlated [135]. When strains of *D. simulans* were crossed, the pure species F_1_ females were then crossed *D. melanogaster *males and the crossability was compared to the two parental strains. Mixed results were found: while one study found that F_1_ females always showed greater levels of hybridization [87], another study found that in most cases F_1_ females showed significantly lower levels of hybridization [86], making it unclear whether increased discrimination within *D. simulans* against heterospecifics is dominant or not. Further inconsistencies include one study that found that X and the third chromosome act additively to contribute to the rejection of *D. melanogaster* males by *D. simulans* females [87], while another study found that the X and the left arm of the second chromosome influenced the trait [133]. These results may be due to the low genetic variability within inbred laboratory lines, and may support the hypothesis that the genetic basis of behavioral isolation varies among populations of the same species. Recently, the right arm of the third chromosome (3R) was mapped using deficiency mapping, revealing five regions (all in areas of low recombination) that contribute to the rejection behavior of *D. simulans* females towards a courting *D. melanogaster* male [35]. While a list of candidate genes in these regions was generated, fine mapping of these regions to the individual gene level remains.

### 6.7. M and Z Forms of *D. melanogaster*

*Drosophila melanogaster* are found all over the world, usually commensally with humans, and it was once thought that there was gene flow between populations, including those found spread across large continents [136]. However, a Zimbabwe population was found to have twice the amount of genetic variation compared to North American populations, with certain variants only present in Zimbabwe [137]. Females from these Zimbabwe lines (Z) show behavioral isolation against males from cosmopolitan regions (M): when they have the choice, Z females prefer to mate with Z males, but show no postzygotic isolation (hybrid sterility or inviability) when they are mated with M males. Females from cosmopolitan regions also show behavioral isolation with Z males, but it is weaker than that seen in Z females [90]. The genetic basis for this strong preference in Z females was mapped to all three major chromosomes, with the largest effect being contributed by the third chromosome [91]. With the use of recombinant lines and visible markers (dominant mutations to identify which homologous chromosome was inherited from which parental species), the genetic basis of the female preference in Z females for Z males was mapped to four regions: a region of large effect and a region of minor effect on the left arm of the third chromosome (3L) and a region that most likely houses two loci on the right arm of the third chromosome (3R) [92].

## 7. Conclusions

In the quest to identify the genetic basis of behavioral isolation, genomic regions have been mapped for interspecific female receptivity in a variety of species pairs. These efforts have yielded maps that vary in refinement from whole chromosomes, chromosomal arms, subchromosomal regions, to specific QTLs. Although the genetic basis of female discrimination may be species pair specific [135], one common attribute of these loci is their location in the genome: most of these loci fall within areas of low recombination, such as species inversion polymorphisms, regions near the centromere, and regions near the telomere. Behavioral isolation loci between *D. santomea* and *D. yakuba* were found near the centromere on 3R [7], and near the telomere for both the *D. simulans* and *D. mauritiana* species pair [6] and the M and Z forms of *D. melanogaster* [92]. Loci responsible for the behavioral isolation between *D. ananassae* and *D. pallidosa* [121], and the isolation between *D. pseudoobscura* and *D. persimilis* [84] all fell within interspecific inversion polymorphisms. Although this was not true for the regions responsible for increased behavioral isolation caused by reinforcement in the latter species pair [109], these loci for reinforcement were not confirmed by further studies [124]. 

Inversions have also been shown to play a role in within-species assortative mating. Unlike other species of *Drosophila*, *D. ananassae* males have spontaneous meiotic recombination which contributes to the entire species having a high degree of inversion polymorphisms. One inversion, called “alpha,” is a large paracentric inversion covering the majority of 2L (3R in *D. melanogaster*). To investigate whether this inversion could contribute to behavioral isolation within this species, Nanda and Singh [120] created karyotypically different strains homozygous for one of three naturally occurring inversions. Through mate choice assays, they found a preference for homogamic matings in all three populations. 

Genomic rearrangements, centromeric, and telomeric areas can act as an island of low recombination between two potentially interbreeding populations, allowing for the creation and maintenance of population-specific gene complexes (genes inherited together). Over time, new mutations can occur within theses complexes and, due to reduced recombination [138], can create a population-typical phenotype if the complexes contain variants for local adaptation [139]. Therefore, even in the face of gene flow between the two groups, a new population identity can be created.

While it has been shown that similar sensory systems may be used for both intra- and interspecific mate discrimination, it is unknown whether these two levels of discriminatory behavior have the same genetic basis. Genomic regions identified as influencing species-specific female preference could contain genes that affect either the auditory or olfactory system, as both are used in mate discrimination, or the brain where this information is processed. If these genes could tolerate a genetic variant causing a slight change in function, selection could then act directly on a new allele, or on other genes within this genetic island, to cause different alleles to reach a high frequency in different populations, causing a slight difference in female mating preference between them. If mutations that occur within these regions cause a change in female preference by influencing assortative mating within species [120], these areas can influence behavioral isolation between species, and thus potentially induce a speciation event [84, 140].

 The genetic basis of interspecific female preference is a significant component necessary for understanding the genetic basis of species isolation. While many broad-scale mapping studies have allowed for a solid understanding of the genetic architecture underlying female preference—the number and relative location of genomic regions contributing to female discrimination—to date, no individual genes for this trait have been identified. This limits the ability to assess the mechanism by which females process and evaluate heterospecific mating signals, and thus maintain species isolation. As the genetic tools available in *D. melanogaster* become more widely available in other systems, and as new mapping techniques are developed, refined genetic dissection of this trait is becoming more tenable. By identifying the genetic mutations that cause interspecific variation in mating behavior, we can start to understand the biological basis species isolation, and better our understanding on the definition of a species. Perhaps the most interesting aspect, however, is that we can finally begin to understand the molecular basis of sex.

## Supplementary Material

Supplementary Table 1 is a comprehensive list of genes that have been shown to have an effect on Drosophila melanogaster's courtship or copulatory behaviour. Each gene's cytological location, molecular function, and effect on behaviour is listed, if known.

## Figures and Tables

**Table 1:** Summary of existing genetic analyses of *Drosophila* species pairs that are behaviorally isolated. The current mode of isolation, trait studied, experimental design (E D), and number of loci potentially affecting behavioral isolation are listed. E D's are chromosome substitution (C), deficiency complementation mapping (D), complementation mapping of single genes (G), homozygous for a mutation (H), introgression (I), microarray (M), quantitative trait locus mapping (Q), and recombination mapping (R).

Species pair	Isolation	Trait	E D	Number of loci
*D. melanogaster* (two “races”)	Allopatric	Male prezygotic isolation [90–92]	C, I	≥5
		Female prezygotic isolation [90–93]	C, I, M	≥4
		Female pheromone production [94]	R	1

*D. melanogaster* and *D. simulans *	Sympatric	Female pheromone production [95]	D	≥5

*D. simulans* and *D. sechellia *	Allopatric	Female pheromone production [56, 74]	Q	≥11
		Male prezygotic isolation [59]	Q	≥1
		Male copulation duration [59]	Q	≥1
		Male genital morphology [96]	Q	≥7–11
		Male sex comb tooth number [96]	Q	≥4
		Male pheromone production [59, 97, 98]	Q, C	≥1–5
		Female prezygotic isolation [2]	C	≥2
		Male courtship song [99]	Q	≥6

*D. simulans* and *D. mauritiana *	Allopatric	Male prezygotic isolation [2, 4, 100]	C	≥2
		Male copulation duration [100]	C	≥3
		Male sex comb tooth number [101]	Q	≥2
		Male genital morphology [101, 102]	Q	≥9
		Female prezygotic isolation [2, 13, 103]	C	≥3
		Mau female discrimination [6]	Q	≥7
		Sim male trait [6]	Q	≥3
		Mau male trait [6]	Q	≥6

*D. mauritiana* and *D. sechellia *	Allopatric	Female pheromone production [104]	R	≥6

*D. mojavensis* (different populations)	Allopatric	Male courtship success [105]	Q	≥1
		Male copulation latency [105]	Q	≥3

*D. mojavensis* and *D. arizonae *	Sympatric	Male prezygotic isolation [106]	C	≥2
		Female prezygotic isolation [106]	C	≥2

*D. heteroneura* and *D. silvestris *	Sympatric	Male head shape [107, 108]	C	≥9-10

*D. pseudoobscura* and *D. persimilis *	Sympatric	Female prezygotic isolation and reinforcement [109, 110]	Q, I	≥4
		Male prezygotic isolation [111, 112]	C, R	≥3
		Male courtship song [113]	Q	≥2-3
		Female prezygotic isolation [84]	I	≥2
		Pheromone production [114]	C	≥2

*D. virilis* and *D. littoralis *	Sympatric	Male song production [115]	C	≥3

*D. virilis* and *D. lummei *	Sympatric	Male courtship song [116]	C	≥4
		Male pheromone productions [117]	C	≥5
		Female pheromone productions [117]	C	≥4

*D. virilis* and *D. a. texana *	Sympatric	Male prezygotic isolation [118]	Q	≥1

*D. virilis* and *D. novamexicana *	Sympatric	Male prezygotic isolation [118]	Q	≥1

*D. auraria* and *D. biauraria *	Sympatric	Male courtship song [55]	C	≥2

*D. montana* (different strains)	Sympatric	Male pheromone production [119]	Q	≥9

*D. santomea *and *D. yakuba *	Sympatric	Female prezygotic isolation [7]	Q	≥3
		Male trait [7]	Q	≥3

*D. ananassae* (different populations)	Sympatric	Assortative mating [120]	H	≥1

*D. ananassae* and *D. pallidosa *	Sympatric	Female prezygotic isolation [3, 121]	C, I, R	≥2
		Male song production [122]	C	≥2
