# Supplementary material for: The Genetic Basis of Female Mate Preference and Species Isolation in Drosophila
Source: Int J Evol Biol. 2012 Aug 23;2012:328392. doi: 10.1155/2012/328392 (PMC3432541; doi:10.1155/2012/328392)
Supplement: Supplementary file 1 — Supplementary Table 1 is a comprehensive list of genes that have been shown to have an effect on Drosophila melanogaster's courtship or copulatory behaviour. Each gene's cytological location, molecular function, and effect on behaviour is listed, if known. [file 328392.f1.docx]

**Supplementary Table 1:**  An alphabetical list of genes shown to have an effect on *Drosophila melanogaster*’s courtship or copulatory behavior. Only genes that have a known genomic location are listed. C.L. = cytological location in *D. melanogaster*. Molecular functions from The Flybase Consortium. All descriptions refer to the affect on male behavior, unless otherwise noted.

| **Name** | **Abbr.** | **C. L.** | **Molecular Function** | **Behavior** | Description |
| --- | --- | --- | --- | --- | --- |
| *a la Voila et a la vapeur* | *Voila* | 86E2-4 | unknown | Courtship behavior | Male-male courtship^[1, 2]^ |
| *Accessory gland-specific peptide 26Aa* | *Acp 26Aa* | 26A1 | hormone | Postmating behavior | Male compound that acts in the female to stimulate the release of oocytes by ovaries^[3]^ |
| *Accessory gland-specific peptide 26Ab* | *Acp 26Ab* | 26A1 | hormone | Postmating behavior | Male compound that reduces female receptivity; resists displacement of sperm by subsequent sperm^[4]^ |
| *Accessory gland-specific peptide 32CD* | *Acp32CD* | 32C1 | hormone | Female receptivity | Male compound that reduces female receptivity^[5]^ |
| *Accessory gland-specific peptide 33A* | *Acp33A* | 33A | hormone | Female receptivity | Male compound that reduces female receptivity ^[5]^ |
| *Accessory gland-specific peptide 36DE* | *Acp36DE* | 36F3 | hormone | Postmating behavior | Resists displacement of sperm by subsequent sperm in the female reproductive tract.^[4, 6]^ |
| *Accessory gland-specific peptide 53Ea* | *Acp 53Ea* | 53D6 | hormone | Postmating behavior | Resists displacement of sperm by subsequent sperm in the female reproductive tract.^[4]^ |
| *Accessory gland-specific peptide 70A* | *Acp70A* | 70A4 | hormone | Female receptivity | Male compound that reduces female receptivity, stimulates oviposition^[7, 8]^ |
| *Accessory gland-specific peptide 98AB* | *Acp98AB* | 98B1 | hormone | Female receptivity | Male compound that reduces female receptivity ^[5]^ |
| *amnesiac* | *amn* | 18F4-19A2 | neuropeptide hormone | Conditioning;  memory | Reduced female song memory;^[9]^ males not conditioned by courtship of fertilized females^[10, 11]^ |
| *apterous* | *ap* | 41F8 | zinc ion binding; specific RNA polymerase II activity | Courtship behavior; Female receptivity | Reduced courtship behavior;^[12, 13]^ reduced female receptivity^[13, 14]^ |
| *bifid*  (aka *optimotor blind*) | *bi* | 4C3-4 | transcription factor; RNA polymerase II transcription factor; transcription regulator | Courtship behavior | Reduced ability to track females^[15, 16]^ |
| *Btk family kinase at 29A*  (aka *fickleP*) | *Btk29A* | 29A1-3 | protein tyrosine kinase; receptor signaling protein tyrosine kinase | Copulation | Reduced copulation duration^[17]^ |
| *Calcium calmodulin kinase II* | *CaMKII* | 102F6-7 | protein serine/threonine kinase; calmodulin binding; calcium/calmodulin-dependent protein kinase | Courtship behavior | Males not conditioned by courtship of fertilized females^[18]^ |
| *cabbage* | *cab* | 11A2-3 | unknown | Courtship behavior | Reduced courtship behavior^[19]^ |
| *cacophony* | *cac* | 10F7-11A1 | voltage-gated calcium channel | Courtship behavior; song | Abnormal song pulse^[20, 21]^; courtship defective^[20]^ |
| *celibate* | *cel* | 12E | unknown | Copulation | Males court but do not copulate^[22]^ |
| *CG9897* |  | 59C1 | serine endopeptidase | Female receptivity | Female propensity to remate^[23]^ |
| *chaste* | *chaste* | 54B1-5 | unknown | Female receptivity | Reduced female receptivity^[24]^ |
| *coitus interruptus* | *coi* | 7D | unknown | Courtship behavior; Copulation | Reduced courtship behavior;^[22]^ reduced copulation duration^[22]^ |
| *courtless* | *crl* | 14F1 | ubiquitin-conjugating enzyme | Courtship behavior | Reduced courtship behavior^[25, 26]^ |
| *croaker* | *cro* | 45E | unknown | Courtship behavior;  Song | Reduced mating success;^[27]^ aberrant courtship song^[27]^ |
| *cuckold* | *cuc* | 27F6 | unknown | Courtship behavior | Males cannot court or mate^[28]^ |
| *curved* | *c* | 52D3-7 | unknown | Courtship behavior | Reduced courtship behavior^[29]^ |
| *desaturase 2* | *desat2* | 87B10 | stearol-CoA desaturase activity | Pheromones | Removes isolation between two populations^[30]^ |
| *dissatisfaction* | *dsf* | 26A1-2 | ligand-dependant nuclear receptor; transcription factor; steroid hormone receptor | Courtship behavior;  Female receptivity | Reduced female receptivity to males with this mutation;^[31, 32]^ defective male abdominal curling;^[32]^  male-male courtship^[31, 32]^ |
| *don giovanni* | *dg* | 5C2 | unknown | Conditioning | Males not conditioned by courtship of fertilized females^[33]^ |
| *Dopa decarboxylase* | *Ddc* | 37C1 | aromatic-L-amino acid decarboxylase | Conditioning | Males not conditioned by courtship of fertilized females^[34]^ |
| *doublesex* | *dsx* | 84E5-6 | DNA binding activity; transcription factor; RNA polymerase II transcription factor; specific RNA poly. II transcription factor; mRNA binding activity; zing ion binding | Courtship behavior; pheromones;  Song; Female receptivity | Defective female pheromone production;^[35]^ defective male courtship and song;^[36]^ male-male courtship;^[36]^ Males induce increased female receptivity.^[37]^ |
| *Ductus ejaculatorius peptide 99B* | *Dup99B* | 99B8 | Unknown | Female receptivity | Male compound that reduces female receptivity;^[38]^ stimulates oviposition^[38]^ |
| *dunce* | *dnc* | 3C9-D1 | cAMP-specific phosphodiesterase; 3’,5’-cyclic-nucleotide phosphodiesterase | Female receptivity;  Memory | Reduced female song memory;^[39, 40]^ increased female mating^[39-41]^ |
| *ebony* | *e* | 93C7-D1 | beta-alanyl-dopamine synthase | Courtship behavior; Song | Reduced courtship behavior;^[12]^ reduced mating success;^[42]^ abnormal song^[43]^ |
| *ether a go-go* | *eag* | 13A2-5 | voltage-gated potassium channel | Courtship behavior;  Conditioning | Males not conditioned by courtship of fertilized females;^[44]^ reduced courtship behavior^[44]^ |
| *flamenco* | *flam* | 20A1-2 | unknown | Courtship behavior | Reduced courtship and altered sequence of behaviors^[45]^ |
| *Fmr1* | *Fmr1* | 85F10-12 | mRNA binding activity; RNA binding activity | Courtship behavior | Reduced courtship behavior^[46]^ |
| *freeze* | *fez* | 22D | unknown | Courtship behavior | Reduced courtship behavior^[47, 48]^ |
| *fruitless* | *fru* | 91A7-B3 | Zinc finger family transcription factor; RNA polymerase II transcription factor | Courtship behavior;  Song | Abnormal song production;^[49, 50]^ defective male abdominal curling;^[51]^ reduced courtship;^[52]^ male-male courtship^[52]^ |
| *he’s not interested* | *hni* | 89E7-90A7 | unknown | Courtship behavior | Reduced courtship behavior^[53, 54]^ |
| *icebox* | *ibx* | 7E10-8A5 | unknown | Female receptivity | Reduced female receptivity^[55]^ |
| *inactive* | *iav* | 6D3 | ion channel | Courtship behavior;  Female receptivity;  Conditioning | Reduced courtship behavior;^[56]^ Reduced female receptivity;^[57]^ males not conditioned by courtship of fertilized females^[57]^ |
| *Intersex* | *ix* | 47F5 | protein binding | Courtship behavior | Reduced courtship behavior ^[58]^ |
| *lingerer* | *lig* | 44A4 | unknown | Courtship behavior;  Copulation | Reduced courtship behavior;^[59]^ Reduced frequency of copulation^[59]^ |
| *no on or off transient A*  (aka *dissonance*) | *nonA* | 14B18-C1 | RNA binding; pre-mRNA splicing factor; poly-pyrimidine tract binding | Courtship behavior;  Song | Reduced mating success;^[60, 61]^ aberrant courtship song^(20, 50, 62-64]^ |
| *Odorant-binding protein 56a* | *Obp56a* | 56E2 | odorant binding | Female receptivity | Female propensity to remate^[23]^ |
| *pale* | *ple* | 65C3 | tyrosine 3-monooxygenase | Courtship behavior | Abnormal courtship behavior^[65, 66]^ |
| *paralytic* | *para* | 14D1-16A2 | voltage-gated sodium channel | Courtship behavior;  Song;  Female receptivity;  Conditioning | Reduced mating success;^[67-70]^ aberrant courtship song;^[29]^  Reduced female receptivity;^[16, 67, 69]^  males not conditioned by courtship of fertilized females^[71]^ |
| *period* | *per* | 3B1-2 | transcription co-repressor; transcription cofactor | Courtship behavior;  Song | Defective courtship song,^[72-76]^ reduced courtship success^[39, 77]^ |
| *quick-to-court* | *qtc* | 25C3-4 | unknown | Courtship behavior | Quick initiation of courtship in males;^[77]^ male-male courtship^[78]^ |
| *Rutabaga* | *rut* | 12F4-5 | adenylate cyclase; calcium/calmodulin-responsive adenylate cyclase | Courtship behavior;  Conditioning;  Female memory | Reduced courtship behavior;^[79]^ Females have reduced song memory;^[9]^ males not conditioned by courtship of fertilized females^[9, 79]^ |
| *sarah* | *sra* | 89B7 | protein binding | Female receptivity | Female propensity to remate^[80]^ |
| *Sex lethal* | *Sxl* | 6F3-5 | RNA binding activity; pre-mRNA splicing factor; translation repressor; nucleic acid binding; mRNA 5’ UTR binding | Courtship behavior; Pheromones | Reduced courtship behavior;^[81]^ Females synthesize inhibitory pheromones^[81]^ |
| *Shaker* | *Sh* | 16F3-6 | voltage-gated potassium channel | Conditioning | Males not conditioned by courtship of fertilized females^[44]^ |
| *slowpoke* | *slo* | 96A14-17 | calcium-activated potassium channel | Song | Aberrant courtship song^[29]^ |
| *spinster* | *spin* | 52E6-7 | membrane protein | Female receptivity | Reduced female receptivity^[82, 83]^ |
| *takeout* | *to* | 96C7 | unknown | Courtship behavior | Reduced courtship behavior^[84]^ |
| *tan* | *t* | 8D1 | beta-alanyl-dopamine hydrolase | Courtship behavior | Reduced courtship behavior^[15, 16]^ |
| *tapered* | *ta* | 46C3-11 | unknown | Courtship behavior | Reduced courtship behavior^[85, 86]^ |
| *technical knockout* | *tko* | 3A3 | structural constituent of ribosome | Courtship behavior | Reduced courtship success^[87, 88]^ |
| *timeless* | *tim* | 23F6 | interacts with Per | Song | Defective courtship song^[76]^ |
| *transformer* | *tra* | 73A10 | pre-mRNA splicing factor | Courtship behavior; Song; | Reduced courtship behavior;^[89-91]^ abnormal song;^[20]^ male-male courtship^[31, 89-91]^ |
| *transformer 2* | *tra2* | 51B6 | RNA binding activity | Female receptivity | Reduced female receptivity^[48]^ |
| *turnip* | *tur* | 18A5-D1 | unknown | Conditioning | Males impaired in conditioning after courting fertilized females^[19, 79]^ |
| *Ubiquitin-conjugating-enzyme-47D* | *Ubc47D* | 47D | ubiquitin conjugating enzyme | Courtship behavior | Defective male courtship behavior^[93]^ |
| *white* | *w* | 3B6 | ATP-binding cassette (ABC) transporter; eye pigment precursor transporter; transmembrane receptor | Courtship behavior | Reduced courtship behavior;^[94, 95]^ male-male courtship^[95]^ |
| *yellow* | *y* | 1A5 | unknown | Mating success | Reduced male mating success^[96, 97]^ |

**References**

[1] Balakireva, M., Stocker, R. F., Gendre, N., and Ferveur, J.-F. (1998). *Voila*, a new *Drosophila* courtship variant that affects the nervous system: behavioral, neural, and genetic characterization. J. Neurosci. 18(1):4335-4343.

[2] Grosjean, Y., Balakireva, M., Dartevelle, L. and Ferveur, J.-F. (2001) *P*Gal4 excision reveals the pleiotropic effects of *Voila*, a *Drosophila* locus that affects development and courtship behaviour. Genet. Res. 77(3):239-250.

[3] Heifetz, Y., Lung, O., Frongillo, E. A. Jr. and Wolfner, M. F. (2000) The *Drosophila* seminal fluid protein Acp26Aa stimulates release of oocytes by the ovary. Curr. Biol. 10(2):99-102.

[4] Clark, A. G., Aguade, M., Prout, T., Harshman, L. G. and Langley, C.H. (1995) Variation in sperm displacement and its association with accessory gland protein loci in *Drosophila* *melanogaster*. Genetics 139(1):189-201.

[5] Wolfner, M. F., Harada, H. A., Bertram, M. J., Stelick, T. J., Kraus, K. W., Kalb, J. M., Lung, Y. O., Neubaum, D. M., Park, M. and Tram, U. (1997) New genes for male accessory gland proteins in *Drosophila* *melanogaster*. Insect Biochem. Molec. Biol. 27(10):825-834.

[6] Lung, Y. O. and Wolfner, M. F. (2001) Identification and characterization of the major *Drosophila* *melanogaster* mating plug protein. Insect Biochem. Molec. Biol. 31(6-7):543-551.

[7] Fleischmann, I., Dauwalder, B., Chapman, T., Cotton, B. and Kubli, E. (1995) Analysing the sex-peptide reaction-cascade in *Drosophila* *melanogaster* using brain mutants. J. Neurogenet. 10(1):26-27.

[8] Nakayama, S., Kaiser, K. and Aigaki, T. (1997) Ectopic expression of sex-peptide in a variety of tissues in *Drosophila* females using the *P*[GAL4] enhancer-trap system. Molec. gen. Genet. 254(4): 449-455.

[9] Kyriacou, C. P. and Hall, J. C. (1984) Learning and memory mutations impair acoustic priming of mating behaviour in *Drosophila*. Nature 308(5954):62-65.

[10] Ackerman, S. L. and Siegel, R. W. (1986) Chemically reinforced conditioned courtship in *Drosophila*: Responses of wild-type and the *dunce*, *amnesiac* and *don giovanni* mutants. J. Neurogenet. 3(2):111-123.

[11] Siegel, R. W. and Hall, J. C. (1979) Conditioned responses in courtship behavior of normal and mutant *Drosophila*. Proc. Natl. Acad. Sci. USA 76:3430-3434.

[12] Crossley, S. and Zuill, E. (1970) Courtship behaviour of some *Drosophila* *melanogaster* mutants. Nature 225:1064-1065.

[13] Ringo, J., Werczberger, R. and Segal, D. (1992) Male sexual signaling is defective in mutants of the *apterous* gene of *Drosophila* *melanogaster*. Behav. Genet. 22:469-487.

[14] Ringo, J., Werczberger, R., Altaratz, M. and Segal, D. (1991) Female sexual receptivity is defective in juvenile hormone-deficient mutants of the *apterous* gene of *Drosophila* *melanogaster*. Behav. Genet. 21(5):453-469.

[15] Cook, R. (1980) The extent of visual control in the courtship tracking of *Drosophila* *melanogaster*. Biol. Cybern. 37(1):41-51.

[16] Tompkins, L., Gross, A. C., Hall, J. C., Gailey, D. A. and Siegel, R. W. (1982) The role of female movement in the sexual behavior of *Drosophila melanogaster*. Behav. Genet. 12:295-307.

[17] Baba, K., Takeshita, A., Majima, K., Ueda, R., Kondo, S., Juni, N. and Yamamoto, D. (1999) The *Drosophila* *Bruton's tyrosine kinase* (*Btk*) homolog is required for adult survival and male genital formation. Molec. Cell. Biol. 19(6):4405-4413.

[18] Joiner, M.A. and Griffith, L. C. (1997) CaM kinase II and visual input modulate memory formation in the neuronal circuit controlling courtship conditioning. J. Neurosci. 17(23): 9384-9391.

[19] Gailey, D. A., Jackson, R., and Siegel, R. W. (1982) Male courtship in *Drosophila*: the conditioned response to immature males and its genetic control. Genetics 102:771-782.

[20] Bernstein, A. S., Neumann, E., Hall, J. C. (1992) Temporal analysis of tone pulses within the courtship songs of two sibling *Drosophila* species, their interspecific hybrid, and behavioral mutants of *Drosophila melanogaster* (Diptera: Drosophilidae). J. Insect Behav. 5(1):15-36.

[21] von Schilcher, F. (1976). The behavior of *cacophony*, a courtship song mutant in *Drosophila melanogaster*. Behav. Biol. 17:187-196.

[22] Billeter, J. C., Goodwin, S. F. and O'Dell, K.M. (2002) Genes mediating sex-specific behaviors in *Drosophila*. Adv. Genet. 47:87-116.

[23] Giardina, T. J., Beavis, A., Clark., A. G. and Fiumera, A. C. (2011) Female influence on pre- and post-copulatory sexual selection and its genetic basis in *Drosophila melanogaster*. Mol. Ecol. 20:4098-4108.

[24] Yamamoto, D., Jallon, J.-M. and Komatsu, A. (1997). Genetic dissection of sexual behavior in *Drosophila melanogaster.* A. Rev. Ent. 42:551-585.

[25] Orgad, S., Rosenfeld, G., Smolikove, S., Polak, T. and Segal, D. (1997) Behavioral analysis of Drosophila mutants displaying abnormal male courtship. Invert Neurosci. 3(2-3):175-83.

[26] Orgad, S., Rosenfeld, G., Greenspan, R. J. and Segal, D. (2000) *courtless*, the *Drosophila* UBC7 homolog, is involved in male courtship behavior and spermatogenesis. Genetics 155(3):1267-1280.

[27] Yokokura, T., Ueda, R., and Yamamoto, D. (1995). Phenotypic and molecular characterization of *croaker*, a new mating behavior mutant of *Drosophila melanogaster*. Jpn. J. Genet. 70:103-117.

[28] Castrillon, D. H., Gnczy, P., Alexander, S., Rawson, R., Eberhart, C. G., Viswanathan, S., and Wasserman, S. A. (1993). Toward a molecular genetic analysis of spermatogenesis in *Drosophila melanogaster*: characterization of male-sterile mutants generated by single *P* element mutagenesis. Genetics 135:489-505.

[29] Peixoto, A. A. and Hall, J. C. (1998) Analysis of temperature-sensitive mutants reveals new genes involved in the courtship song of *Drosophila*. Genetics 148(2):827-838.

[30] Takahashi, A., Tsaur, S.-C., Coyne, J. A. and Wu, C.-I. (2001). The nucleotide changes governing cuticular hydrocarbon variation and their evolution in *Drosophila melanogaster*. Proc. Natl. Acad. Sci. USA 98(7):3920-3925.

[31] Finley, K. D., Taylor, B. J., Milstein, M., and McKeown, M. (1997). *dissatisfaction*, a gene involved in sex-specific behavior and neural development of *Drosophila melanogaster*. Proc. Natl. Acad. Sci. USA 94:913-918.

[32] Finley, K. D., Edeen, P. T., Foss, M., Gross, E., Ghbeish, N., Palmer, R. H., Taylor B. J., McKeown, M. (1998) *dissatisfaction* encodes a tailless-like nuclear receptor expressed in a subset of CNS neurons controlling *Drosophila* sexual behavior. Neuron. Dec;21(6):1363-74.

[33] Gailey, D. A. and Siegel, R. W., (1989) A mutant strain in *Drosophila* *melanogaster* that is defective in courtship behavioral cues. Anim. Behav. 38(1):163-169.

[34] Tempel, B. L., Livingstone, M. S. and Quinn, W. G. (1984) Mutations in the *dopa decarboxylase* gene affect learning in *Drosophila*. Proc. Natl. Acad. Sci. USA 81:3577-3581.

[35] Jallon, J.-M. (1984). A few chemical words exchanged by *Drosophila* during courtship and mating. Behav. Gen. 14(5):441-477.

[36] Arthur, B. I. Jr., Jallon, J.-M., Caflisch, B., Choffat, Y. and Nothiger, R. (1998) Sexual behavior in *Drosophila* is irreversibly programmed during a critical period. Curr. Biol. 8(21):1187-1190.

[37] Villella, A. and Hall, J. C. (1996). Courtship anomalies caused by the *doublesex* mutations in *Drosophila melanogaster*. Genetics 143:331-344.

[38] Saudan, P., Hauck, K., Soller, M., Choffat, Y., Ottiger, M., Sporri, M., Ding, Z., Hess, D., Gehrig, P. M., Klauser, S., Hunziker, P. and Kubli, E. (2002) *Ductus* *ejaculatorius peptide 99B* (*DUP99B*), a novel *Drosophila* *melanogaster* sex-peptide pheromone. Europ. J. Biochem. 269(3):989-997.

[39] Kyriacou, C. P. (1990) The molecular ethology of the *period* gene in *Drosophila*. Behav. Genet. 20:191-211.

[40] Kyriacou, C. P. and Hall, J. C. (1985) Action potential mutations stop a biological clock in *Drosophila*. Nature 314(6007):171-3.

[41] Hall, J. C. (1984) Complex brain and behavioral functions disrupted by mutations in *Drosophila*. Dev. Genet. 4:355-378.

[42] Rendel, J. M. (1951) Mating of *ebony* vestigial and wild type *Drosophila* *melanogaster* in light and dark. Evolution 5:226-230.

[43] Kyriacou, C. P., Burnet, B. and Connolly, K. (1978) The behavioural basis of overdominance in competitive mating success at the *ebony* locus of *Drosophila* *melanogaster*. Anim. Behav. 26:1195-1206.

[44] Cowan, T. M. and Siegel, R. W., (1984) Mutational and pharmacological alterations of neuronal membrane function disrupt conditioning in *Drosophila*. J. Neurogenet. 1(4):333-344.

[45] Romanova, L. G., Romanova, N. I., Subocheva, E. A. and Kim, A. I. (2000) [Mating success and courtship ritual in strains of *Drosophila* *melanogaster* carrying mutation *flamenco*.] Genetika, Moscow 36(4):500-504.

[46] Dockendorff, T. C., Su, H. S., McBride, S. M., Yang, Z., Choi, C. H., Siwicki, K. K., Sehgal, A. and Jongens, T. A.. (2002) *Drosophila* Lacking *dfmr1* activity show defects in circadian output and fail to maintain courtship interest. Neuron 34(6):973-984.

[47] Nitasaka, E. (1995) Molecular analysis of mating behavior mutation, *freeze* in *Drosophila* *melanogaster*. Jpn J. Genet. 70(6):740.

[48] Nitasaka, E. and Yamazaki, T. (1994) Isolation of mating behavioral mutations in *Drosophila* *melanogaster*. Jpn J. Genet. 69(6):784.

[49] Ryner, L. C., Goodwin, S. F., Castrillon, D. H., Anand, A., Villella, A., Baker, B. S., Hall, J. C., Taylor, B. J. and Wasserman, S. A. (1996) Control of male sexual behavior and sexual orientation in *Drosophila* by the *fruitless* gene. Cell.87:1079-1089.

[50] Wheeler, D. A., Kulkarni, S. J., Gailey, D. A. and Hall, J. C. (1989) Spectral analysis of courtship songs in behavioral mutants of *Drosophila melanogaster*. Behav. Genet. 19(4):503-528.

[51] Gailey, D. A., Taylor, B. J. and Hall, J. C. (1991) Elements of the *fruitless* locus regulate development of the muscle of Lawrence, a male-specific structure in the abdomen of *Drosophila melanogaster* adults. Development 113(3):879-890.

[52] Baker, B. S., Taylor, B. J., Hall, J. C. (2001) Are complex behaviors specified by dedicated regulatory genes? Reasoning from *Drosophila*. Cell 105(1):13-24.

[53] Bubis, J. A., Degreen, H. P., Unsell, J. L. and Tompkins, L. (1998) Temporal manipulation of ejaculate components by newly fertilized *Drosophila* *melanogaster* females. Anim. Behav. 55(6):1637-1645.

[54] Friedman, R., Harvey, M., Martin, P., and Tompkins, L. (1995) *hni* results in a behavioral male sterile phenotype. A. Dros. Res. Conf. 36: 89B.

[55] Kerr, C., Ringo, J., Dowse, H. and Johnson, E. (1997) *icebox*, a recessive *X*-linked mutation in *Drosophila* causing low sexual receptivity. J. Neurogenet. 11(3-4):213-229.

[56] O'Dell, K. M., Burnet, B. and Jallon, J.-M. (1989) Effects of the *hypoactive* and *inactive* mutations on mating success in *Drosophila* *melanogaster*. Heredity 62(3):373-381.

[57] O'Dell, K. M. (1993) The effect of the *inactive* mutation on longevity, sex, rhythm and resistance to p-Cresol in *Drosophila* *melanogaster*. Heredity 70(4):393-399.

[58] McRobert, S. P. and Tompkins, L. (1985). The effect of *transformer*, *doublesex*, and *intersex* mutations on the sexual behavior of *Drosophila melanogaster*. Genetics 111:89-96.

[59] Kuniyoshi, H., Baba, K., Ueda, R., Kondo, S., Awano, W., Juni, N. and Yamamoto, D. (2002) *lingerer*, a *Drosophila* gene involved in initiation and termination of copulation, encodes a set of novel cytoplasmic proteins. Genetics 162(4):1775-1789.

[60] Rendahl, K. G., Jones, K. R., Kulkarni, S. J., Bagully, S. H., and Hall, J. C. (1992). The *dissonance* mutation at the *no-on-transient-A* locus of *D. melanogaster*: genetic control of courtship song and visual behaviors by a protein with putative RNA-binding motifs. J. Neurosci. 12:390-407.

[61] Rendahl, K. G. and Hall, J. C. (1996) Temporally manipulated rescue of visual and courtship abnormalities caused by a *nonA* mutation in *Drosophila*. J. Neurogenet. 10(4):247-256.

[62] Krejci, C. M., Rendahl, K. G. and Hall, J. C. (1994) Rescue of behavioral phenotypes by a *nonA*-null transgene is influenced by chromosomal position effects. D. I. S. 75:117.

[63] Kulkarni, S. J., Steinlauf, A. F., and Hall, J. C. (1988). The *dissonance* mutant of courtship song in *Drosophila melanogaster*: isolation, behavior and cytogenetics. Genetics 118:267-285.

[64] Sandrelli, F., Campesan, S., Rossetto, M., Benna, C., Zieger, E., Megighian, A., Couchman, M., Kyriacou, C. P. and Costa, R. (2001) Molecular dissection of the 5' region of *no-on-transientA* of *Drosophila melanogaster* reveals *cis*-regulation by adjacent dGpi1 sequences. Genetics 157(2):765-775.

[65] Buchner, E. (1991). Genes expressed in the adult brain of *Drosophila* and effects of their mutations on behavior: a survey of transmitter- and second messenger-related genes. J. Neurogenet. 7:153-192.

[66] Neckameyer, W. S. (1998) Dopamine modulates female sexual receptiveness in *Drosophila melanogaster*. J. Neurogenet. 12:101-114.

[67] Gailey, D. A., Lacaillade, R. C. and Hall, J. C. (1986) Chemosensory elements of courtship in normal and mutant, olfaction-deficient *Drosophila melanogaster*. Behav. Genet. 16(3):375-405.

[68] Lilly, M., and Carlson, J. (1989). *Smellblind*: a gene required for *Drosophila* olfaction. Genetics 124:293-302.

[69] Markow, T. A. (1987) Behavioral and sensory basis of courtship success in *Drosophila melanogaster*. Proc. Natl. Acad. Sci. USA 84(17): 6200-6204.

[70] Tompkins, L. Hall, J. C. and Hall, L. M. (1980) Courtship-stimulating volatile compounds from normal and mutant *Drosophila*. J. Insect Physiol. 26(10):689-697.

[71] Tompkins, L. Siegel, R. W., Gailey, D. A. and Hall, J. C. (1983) Conditioned courtship in *Drosophila* and its mediation by association of chemical cues. Behav. Genet. 13(6):565-578.

[72] Crossley, S. (1988) Failure to conform rhythms in *Drosophila* courtship song. Anim. Behav. 36(4):1098-1109.

[73] Ewing, A. W. (1988) Cycles in the courtship song of male *Drosophila melanogaster* have not been detected. Anim. Behav. 36(4):1091-1097.

[74] Kyriacou, C. P., and Hall, J. C. (1980). Circadian rhythm mutations in *Drosophila* affect short-term fluctuations in the male’s courtship song. Proc. Natl. Acad. Sci. USA 77:6929-6933.

[75] Kyriacou, C. P. and Hall, J. C. (1986) Interspecific genetic control of courtship song production and reception in *Drosophila*. Science 232(4749):494-497.

[76] Kyriacou, C. P. and Hall, J. C. (1988) Comment on Crossley's and Ewing's failure to detect cycles in *Drosophila* mating. Anim. Behav. 36(4):1110.

[77] Jackson, F. R., Gailey, D. A. and Siegel, R. W. (1983) Biological rhythm mutations affect an experience-dependent modification of male courtship behaviour in *Drosophila melanogaster*. J. Comp. Physiol. 151(4):545-552.

[78] Gaines, P., Tompkins, L., Woodard, C. T., and Carlson, J. R. (2000). *quick-to-court*, a *Drosophila* mutant with elevated levels of sexual behavior, is defective in a predicted coiled-coil protein. Genetics 154:1627-1637.

[79] Gailey, D. A., Jackson, F. R. and Siegel, R. W. (1984). Conditioning mutations in *Drosophila melanogaster* affect an experience-dependent behavioral modification in courting males. Genetics 106:613-623.

[80] Ejima, A., Tsuda, M., Takeo, S., Ishii, K., Matsuo, T. and Aigaki, T. (2004). Expression level of *sarah*, a homolog of *DSCR1*, is a critical for ovultion and female courtship behavior in *Drosophila melanogaster*. Genetics 168:2077-2087.

[81] Tompkins, L. and McRobert, S. P. (1995) Behavioral and pheromonal phenotypes associated with expression of loss-of-function mutations in the *Sex-lethal* gene of *Drosophila* melanogaster. J. Neurogenet. 9(4):219-226.

[82] Nakano, Y., Fujitani, K., Kurihara, J., Ragan, J., Usui-Aoki, K., Shimoda, L., Lukacsovich, T., Suzuki, K., Sezaki, M., Sano, Y., Ueda, R., Awano, W., Kaneda, M., Umeda, M. and Yamamoto, D. (2001) The novel evolutionary conserved *Drosophila* membrane protein *spinster* is required for development of normal sexual receptivity and oogenesis. Mol Cell Biol. 21(11):3775-88.

[83] Suzuki, K., Juni, N., and Yamamoto, D. (1997). Enhanced mate refusal in female Drosophila induced by a mutation in a spinster locus. Appl. Entomol. Zool. 32:235-243.

[84] Dauwalder, B., Tsujimoto, S., Moss, J. and Mattox, W. (2002) The *Drosophila* *takeout* gene is regulated by the somatic sex-determination pathway and affects male courtship behavior. Genes Dev. 16(22):2879-2892.

[85] Bien-Willner, R. D., and Doane, W. W. (1997) 13^th^ Intrn. Congr. Devel. Biol. Abstract 291, Snowbird, USA.

[86] Wood, D.D. and Butterworth, F.M. (1972) Mating behavior and reproductive tract morphology of male-sterile mutants. D. I. S. 49:67-68.

[87] Emmons, S. W. and Lipton, J. (2003) Genetic basis of male sexual behavior. J. Neurobiol. 54(1):93-110.

[88] Toivonen, J. M., O'Dell, K. M., Petit, N., Irvine, S. C., Knight, G. K., Lehtonen, M., Longmuir, M., Luoto, K., Touraille, S., Wang, Z., Alziari, S., Shah, Z. H. and Jacobs, H. T. (2001) *technical knockout*, a *Drosophila* model of mitochondrial deafness. Genetics 159(1):241-254.

[89] Ferveur, J.-F., Savarit, F., O’Kane, C. J., Sureau, G., Greenspan, R. J., and Jallon, J.-M. (1997). Genetic feminization of pheromones and its behavioral consequences in *Drosophila* males. Science 276:1555-1558.

[90] Taylor, B. J., Villella, A., Ryner, L. C., Baker, B. S., and Hall, J.C. (1994). Behavioral and neurobiological implications of sex-determining factors in *Drosophila*. Dev. Genet. 15:275-296.

[91] Waterbury, J. A., Jackson, L. L. and Schedl, P. (1999) Analysis of the *doublesex* female protein in *Drosophila* *melanogaster*. Role In sexual differentiation and behavior and dependence on *intersex*. Genetics 152(4):1653-1667.

[92] O'Dell, K. M. and Kaiser, K. (1995) Functional dissection of the *Drosophila* mushroom bodies by selective feminization of genetically defined subcompartments. Neuron 15(1):55-61.

[93] Orgad, S. and Segal, R. W. (1995) Possible involvement of the Ubiquitin-conjugating-system in courtship behavior in *Drosophila*. J. Neurogenet. 10(1):42.

[94] Sturtevant, A. H. (1915). Experiments in sexual recognition and the problems of sexual selection in *Drosophila*. J. Anim. Behav. 5:351-366.

[95] Zhang, S. D. and Odenwald, W. F. (1995) Misexpression of the *white* (*w*) gene triggers male-male courtship in *Drosophila*. Proc. Natl. Acad. Sci. USA 92(12):5525-5529.

[96] Burnet, B. and Wilson, R., (1980) Pattern mosaicism for behaviour controlled by the *yellow* locus in *Drosophila* *melanogaster*. Genet. Res. 36(3):235-247.

[97] Wilson, R., Burnet, B., Eastwood, L. and Connolly, K. (1976) Behavioural pleiotropy of the *yellow* gene in *Drosophila* *melanogaster*. Genet. Res. 28:75-88.
